# Conservative, Surgical, and Prosthetic Treatment of a Patient with a Periapical Lesion Associated with an Atypical Intraoral Sinus Tract

**DOI:** 10.1155/2015/495206

**Published:** 2015-05-06

**Authors:** Michael Wolgin, Peter Tschoppe, Andrej M. Kielbassa

**Affiliations:** ^1^Centre for Operative Dentistry and Periodontology, University of Dental Medicine and Oral Health, Danube Private University (DPU), Steiner Landstrasse 124, 3500 Krems an der Donau, Austria; ^2^Zahn & Mensch, Private Practice for Interdisciplinary Dentistry, Innrain 6, 6020 Innsbruck, Austria

## Abstract

This report describes a clinical case with an atypical intraoral sinus tract formation from diagnosis and treatment to short-term outcome and definitive prosthetic rehabilitation. In detail, the patient underwent conservative nonsurgical root canal treatment followed by guided bone augmentation of the regions involved in periapical inflammation and sinus tract formation. The removal of the inflammatory source of the lesion as well as the affected tissue clearly led to a healing of the surrounding bone tissues. Subsequently, the tooth was reconstructed using a fibreglass post and a metal-ceramic crown; an implant was successfully placed in the previously inflamed bone region.

## 1. Introduction

Infections of the dental pulp may be caused by caries, operative dental procedures, and traumata; they are always initiated by a mixed, predominantly gram-negative, anaerobic bacterial flora [1]. Initially, pulpal infections lead to a local immune response in the dental pulp tissues, which is often overstrained with the elimination of the invading microorganisms [2]. As a consequence, this infection can lead to a total pulp necrosis, stimulating the secondary immune response in the periapical region [3]. Thus, it may be stated that the microbial colonization of the root canal system has been long established to play a crucial role for the formation of the periapical lesions [4].

Depending on their cellular composition and structural organization, these lesions can be referred to as abscesses, granulomas, or apical cysts. The periapical abscess is a suppurative process, leading to spontaneous pus drainage and, consequently, to the formation of a drainage duct, called fistula or sinus tract [1, 3–5]. Usually, pus accumulates beneath the mucosa, producing a localized swelling, leading to ruptures at later stages, and thus allowing pus to escape into the oral cavity. Intraorally, the opening is usually visible on the attached buccal gingiva or in the vestibule [5]. Occasionally, cutaneous sinus tract or penetration into the maxillary sinus or nasal cavity has been observed and described [6–9]. Thus, the sinus tract may open extraorally anywhere on the face and neck.

After the formation of the sinus tract, typical symptoms of acute periapical inflammation, such as exactly locatable intensive pain, continuously subside and the drainage can persist until the tooth is treated or removed [3–5]. In this case report, a patient with an unusual sinus tract occurrence was successfully cured by means of a conventional root canal treatment. In addition, due to the great extent of alveolar bone resorption, but mainly due to urgency of prosthetic rehabilitation of the patient, guided bone regeneration/augmentation was performed.

## 2. Case Report

### 2.1. Anamnesis

A 58-year-old male patient came for regular dental examination at the Department of Operative Dentistry and Periodontology, University School of Dental Medicine, CharitéCentrum 3, Charité - Universitätsmedizin Berlin. Subjectively, the patient was without any dental complaints. His medical history showed no significant findings. However, his dental history revealed a complicated extraction of the mandibular right first molar performed two years earlier, which had led to a free-end situation (without a definitive rehabilitation up to the first appointment).

### 2.2. Clinical Findings

**Figure 1:**
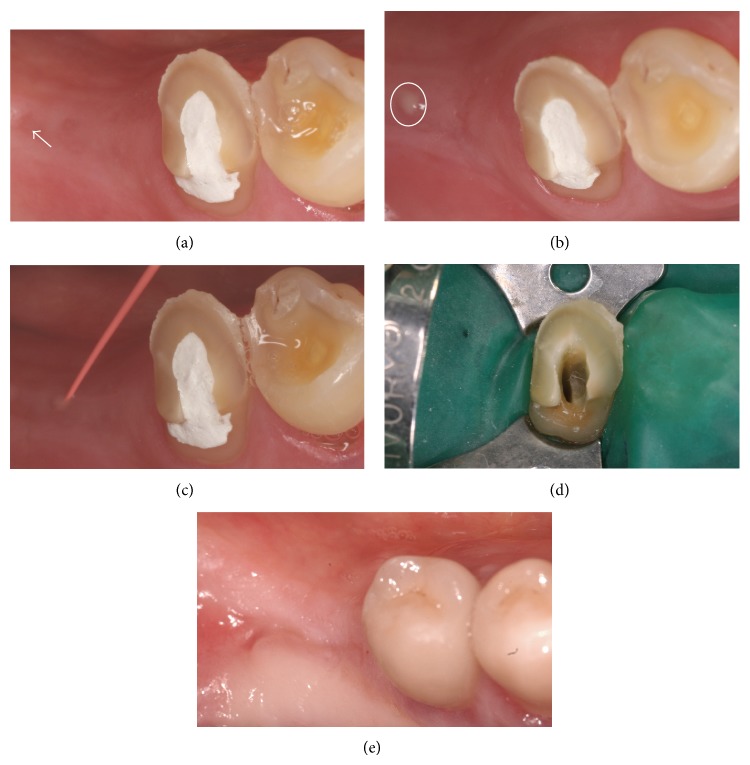
(a) Drained sinus tract on the top side of alveolar crest. (b) Suppuration from the sinus tract. (c) Tracking of the sinus tract with gutta-percha point. (d) Access cavity and rubber dam. (e) Clinical situation after complete healing (18 months after the treatment).

**Figure 2:**
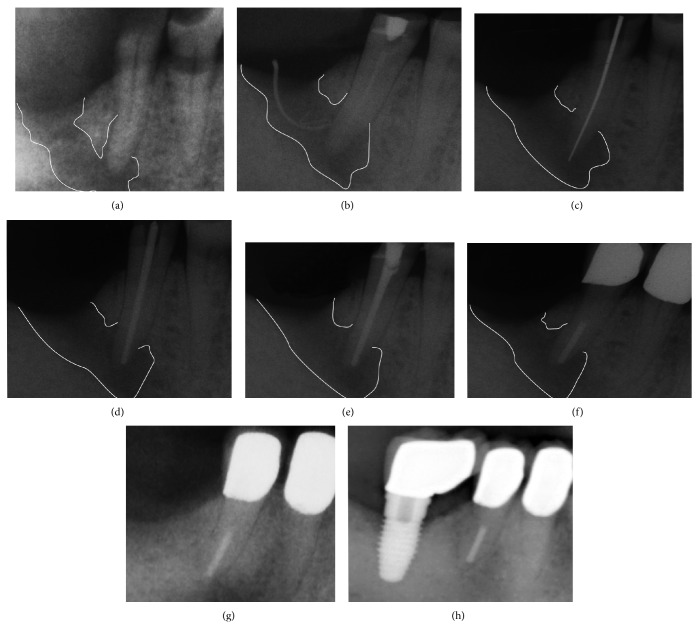
(a) Periapical radiograph prior to root canal treatment. (b) Tracking of the sinus tract with gutta-percha point. (c) Determination of working length. (d) Master point radiograph. (e) Completed root canal treatment. (f) Reconstruction with fibre post and metal-ceramic crown; partial healing of the bone defect. (g) Radiographic situation after complete healing. (h) Radiographic situation of the implant after five years in situ.

Upon examination of the mandibular right second premolar, a carious lesion was detected on the occlusal surface. Pulp sensitivity to cold (−45°C; ORBIS Dental, Munster, Germany) was negative, and the tooth was not sensitive to percussion. At a first glance, there was no evidence to suggest a sinus tract; naked-eye inspection of the typical predilection sites for the sinus tract formation on the buccal and vestibular sides of the alveolar ridge revealed no pathological findings. Therefore, an immanent implantation and prosthetic rehabilitation were discussed and scheduled with the patient. However, a radiographically visible periapical lesion, involving the distal region of the mandible (radiolucent area, see Figure 2(a)), was evident. Accurate examination of the distal attached gingiva revealed a draining sinus tract on the top of alveolar ridge, which suppurated upon digital compression (Figures 1(a) and 1(b)). The sinus tract was explored with a gutta-percha cone to the apical area of the mandibular right second premolar (Figures 1(c) and 2(b)).

### 2.3. Diagnosis

Based on these findings, the patient was diagnosed with an atypical odontogenic intraoral sinus tract secondary to chronic periradicular periodontitis of the mandibular right second premolar (probably, in association with the complicated extraction of mandibular right first molar performed two years earlier).

### 2.4. Therapy

#### 2.4.1. Endodontic Treatment

The endodontic treatment was carried out without any anaesthetics, thus confirming the existence of necrotic pulp tissue. A rubber dam (Coltène Whaledent, Langenau, Germany) was placed and, after caries removal, an access cavity was prepared (Figure 1(d)). On the floor of the pulp chamber one orifice could be detected, which was explored with number 15 file (VDW, Munich, Germany) to clarify the exact localization of the root canal. To determine the length of the root canal, an apex locator (Raypex 5, VDW) was used. By initial placement of a number 15 silver point (VDW), using an individualized X-ray holder, radiographic working length was determined (Figure 2(c)).

The root canal was prepared with rotary files and reamers (Flex Master, VDW) using a combined crown-down and step-back technique under irrigation with 5% sodium hypochlorite (Hedinger, Stuttgart, Germany) and 0.2% chlorhexidine digluconate solution (Charité - Universitätsmedizin Berlin, Germany). The preparation of the root canal was performed to ISO size 35 master point, up to a working length of 15.5 mm (Figure 2(d)) as intracanal antibacterial interim dressing (calcium hydroxide; Ultracal XS; Ultradent, South Jordan, UT, US) was used. Two weeks later, no more suppuration from the sinus tract upon digital compression was observed. Due to possible recontamination from the infected bony lesion [10], the interim dressing was changed twice every two weeks and a judicious curettage of the sinus tract using a small excavator was performed under anaesthesia (Ultracain D-S; Sanofi-Aventis, Frankfurt, Germany). During this period the patient remained asymptomatic; meanwhile upon radiographic investigation the periapical area showed signs of partial bony recovery. Subsequently, the root canal obturation with gutta-percha points (VDW) and resinous sealer (AH Plus; Dentsply Maillefer, Ballaigues, Switzerland) was performed using the lateral condensation technique (Figure 2(e)).

#### 2.4.2. Restorative Procedure

Six weeks later the endodontically treated premolar was reconstructed with a fibreglass post and a composite restoration (Figure 2(f)). Rubber dam (Coltène Whaledent) was placed and the existing temporary composite filling was removed. A cylindrical post space was prepared with a low speed bur (DT Light Post, VDW) provided by the manufacturer, up to a depth of 10 mm of the 15.5 mm root length. The root canal walls were etched with 37% phosphoric acid (OmniDent, Rodgau Nieder-Roden, Germany) for 30 s, followed by irrigation of the root canal with distilled water and thorough drying with absorbent paper points. The self-curing dentine adhesive (Clearfil New Bond, Kuraray, Tokyo, Japan) was applied in two coats using a brush tip and then gently air-dried to avoid a collapse of the dentinal collagen fibrilar network. Additionally, the excess of the dentine adhesive has been removed using the paper points. Prior to post insertion, the post was cleaned using 2-propanol, followed by the application of a silane coupling agent (Monobond S, Ivoclar Vivadent, Schaan, Liechtenstein). The base and catalyst of resin cement were mixed according to the manufacturer's instructions. In order to achieve a uniform, continuous cement layer, the cement was applied onto the post surface as well as into the prepared root canal (post space). The post was inserted into the canal with pumping movements to prevent air entrapment. The access cavity was closed using the self-curing composite (Clearfil Core, Kuraray). Subsequently, the patient underwent prosthetic treatment of his advanced tooth wear (including the described tooth) by means of metal-ceramic crowns (Figures 2(f) and 2(g)).

#### 2.4.3. Surgical Treatment and Implantation

One year later, the patient returned for the follow-up examination. The mandibular right second premolar still was completely asymptomatic. Radiographically, the periapical area showed a partial bony recovery, comparable to the situation several weeks after endodontical treatment (Figure 2(f)). However, the evidence of the former sinus tract could still be recognised. To eliminate the granulation tissue distally to the mandibular right second premolar an access flap was performed, followed by guided bone regeneration/augmentation with the purpose of providing new bone formation.

Immediately before the procedure, the patient rinsed for 2 minutes with a 0.2% chlorhexidine digluconate solution (Chlorhexamed, GlaxoSmithKline Consumer Healthcare, Buehl, Germany). The treatment was initiated by establishing local anesthesia (Ultracain D-S), followed by full thickness mucoperiosteal flap elevation and removal of the compromised elements. The granulation tissue was thoroughly curetted and removed using a surgical curette (Aesculap, Tuttlingen, Germany). After removal of the granulation tissue, a defined 5-wall bony defect remained, which was cleaned manually and by means of sonic instrumentation (Sonicsys, KaVo, Biberach, Germany), followed by copious irrigation with saline. Subsequently, the defect was filled with an alloplastic bone grafting material (Bio-Oss, Geistlich, Wolhusen, Switzerland). A porcine collagen membrane (Bio-Gide, Geistlich) was sized and placed over the defect. The soft tissue was repositioned over the augmented region and sutured with 4.0 silk sutures (Resorba, Nuremberg, Germany). Examination one week later showed uneventful healing without any exposure of the membrane.

The postoperative appointment six months after augmentation showed no remaining evidence of the sinus tract; radiographically, the periapical area showed a complete healing (Figures 1(e) and 2(g)). Subsequently, a 6 mm × 10 mm implant (NobelReplace Tapered Groovy, Nobel Biocare, Zuerich, Switzerland) was inserted in the augmented bone region. Additionally, the treatment success was evaluated in the short recall intervals. The implant remained stable after five years of evaluation (Figure 2(h)).

## 3. Discussion

Sinus tracts developing periapically from chronic low grade inflammations are considered a common clinical observation [5]. However, chronic sinus tracts with atypical localization can present a diagnostic challenge to the clinician [6]. In the present case report an inspection of the typical predilection sites of sinus tract formation, often noted buccally of the alveolar ridge, revealed no pathological findings. However, radiographic and accurate intraoral examination of the distal attached gingiva revealed a draining sinus tract on the top of alveolar ridge. In spite of diagnostic difficulties due to atypical localization of the fistula, the patient could be treated successfully.

The localization of any sinus tract's aperture buccally or vestibularly of the alveolar ridge can be explained by the preferential flow of exudate through less resistant areas, spread through bone marrow, periosteum, and connective tissue and between the fascias and will drain onto the epithelial surface on the buccal or vestibular side of the alveolar ridge [3–5]. In rare cases, sinus tracts will end even extraorally [6–8, 11]. Currently there is no evidence available regarding sinus tract formation on the top of the alveolar ridge. Apparently, such formations could occur as a result of disordered wound healing after tooth extraction, combined with the active inflammation around the neighbouring tooth's root apex. Thus, the suppuration from an area of inflammation could drain into the dental extraction wound and then form a sinus tract with the opening on the top of alveolar ridge.

Sinus tracts of dental origin may be misdiagnosed as periodontal abscesses, osteomyelitis, neoplasms, tuberculosis, or actinomycosis [12]. As dental causes, traumata, retained roots, residual chronic infections of the jaw, and pulp inflammation or necrosis can be considered [12]. In case of a pulpal aetiology, sinus tracts usually respond well to sufficient conservative endodontic therapy, and the prognosis for successful healing of sinus tracts without surgical treatment is very favourable [6–9, 11]. Alternative therapies, such as dental extractions, should undoubtedly be taken into consideration only in nonresponding cases.

The root canal treatment was performed by means of rotary files and reamers using a combined crown-down and step-back technique under irrigation with 5% sodium hypochlorite and 0.2% chlorhexidine digluconate solution. In spite of any strong antibacterial effects of sodium hypochlorite, this rinsing solution will not lead to total absence of bacteria. Even the use of calcium hydroxide as an interim dressing does not exert sufficient antibacterial action against* Enterococcus faecalis* in the root canal [13].* Enterococcus faecalis* seems to be the most prominent microbe in endodontic failures; indeed, this is a bacterium that is very difficult to eliminate using conventional methods. Regarding the elimination of* Enterococcus faecalis*, the root canal rinsing has been shown to be most efficient when sodium hypochlorite is combined with chlorhexidine digluconate [13].

The present case demonstrates a delayed regeneration of the sinus tract in spite of successful endodontic treatment. Previous investigations showed that sinus tracts are usually lined with granulomatous tissue, although, in more advanced stages, an epithelial lining might also be present [14, 15]. Moreover, depending on the extent of tissue damage, a complete repair of lesions may take days to years. Clinical studies reported that labial bone defects measuring 5 to 8 mm in diameter healed within 5 months [14, 16, 17]. In contrast, defects measuring 9 to 12 mm were still filled with avascular fibrous connective tissue up to 8 months following treatment. In case of unsuccessful endodontic treatment and sinus tract curettage, surgical intervention might be necessary [16, 17]. Indeed, the authors of this case report are convinced that the healing would have occurred after a longer observation period; however, due to the urgency of prosthetic rehabilitation of the patient guided bone regeneration/augmentation was performed followed by prosthetic treatment of patient's advanced tooth wear.

Because of the unilateral free-end situation, a restoration, such as a fixed cantilever bridge, or a solitary dental implant was clearly indicated. Cantilever bridges are considered to be a compromise but are preferred to a removable partial denture, especially for unilateral edentulous dentitions. Technical failures are more commonly observed if nonvital teeth are used as abutments for cantilever bridges [18]. In consideration of the antagonistic dentition and the described technical and biological aspects (nonvital tooth), the decision preferred is the insertion of a dental implant. The treatment success has been evaluated in short recall intervals of six months. The implant remained stable after five years of evaluation. Based on these observations the treatment may be considered to have a favorable prognosis.

## 4. Conclusions

In the presented case report the healing of the atypical sinus tract associated with periapical lesion of endodontic origin was observed. However, due to urgency of prosthetic rehabilitation of the patient, guided bone augmentation, implantation, and definitive reconstruction had to be accelerated. In many instances, like with the present case, accurate radiographic and intraoral examinations are of great importance. Precise radiographic examination can alert clinicians to the presence of variations, leading to successful treatment.
